# Effect of planting density and harvesting date on yield and quality of *Artemisia argyi*

**DOI:** 10.7717/peerj.20565

**Published:** 2026-01-13

**Authors:** Haiyan He, Dandan Yang, Lixin Zhang, He Zhang, Chaoze Wang, Nayuan Yao, Yanyan Liu, Zhimin Li, Hongrui Zhang

**Affiliations:** 1 College of Agriculture, Henan Agricultural University, Zhengzhou, China; 2 Henan Zhiyuantang Pharmaceutical Co. Ltd., Nanyang, China

**Keywords:** *Artemisia argyi*, Volatile oil, Moxa yield rate, Eucalyptol, Borneol

## Abstract

**Background:**

Field investigations revealed that during the production of *Artemisia argyi* Lévl. et Vant. excessive planting density and untimely harvesting lead to desiccation and abscission of leaves in the middle and lower plant sections. The height of these withered leaves (dead leaf height) accounted for over 50% of the plant height, significantly reducing both the yield and quality of the medicinal herb *A. argyi* leaves.

**Methods:**

This study employed field experiments using Nanyang *A. argyi* as the test material. Agronomic traits (including plant height, canopy width, number of effective leaves on the main stem, dead leaf height, stem diameter, internode length, and yield) and quality indicators (including volatile oil content, moxa yield rate, eucalyptol content, borneol content, and total flavonoid content) were measured under different planting density treatments and at different harvest times.

**Results:**

The results demonstrated that reducing planting density promoted sturdier plant growth, increased the number of effective leaves on the main stem, decreased the dead leaf height, and increased the dry matter ratio of the *A. argyi* leaves. As the harvest time was delayed, the number of effective leaves on the main stem, dead leaf height, and yield all gradually increased. The volatile oil content, eucalyptol content, and borneol content in *A. argyi* leaves increased gradually with decreasing planting density. These same components initially increased and then decreased as the harvest time was extended. Conversely, the combustion calorific value of *A. argyi* down, total flavonoid content, polysaccharide content, and polyphenol content generally decreased gradually with reduced density. The combustion calorific value, total flavonoid content, and polyphenol content also exhibited an initial increase followed by a decrease with prolonged harvest time.

**Conclusions:**

Comprehensive analysis of essential oil content, down yield rate, eucalyptol content, borneol content, dry matter ratio, and yield across different densities and harvest periods indicated that for spring-planted *A. argyi*, a planting spacing of 20 cm × 40 cm combined with harvesting one week after the Dragon Boat Festival resulted in superior overall quality and higher yield.

## Introduction

*Artemisia argyi* Lévl. et Vant. is a plant in the Asteraceae family (China Pharmacopoeia Committee, 2020). Modern pharmacological studies have demonstrated that *A. argyi* leaves have antibacterial, antiviral, antioxidant, antispasmodic, antitussive, expectorant, hemostatic, sedative, and anti-allergy effects (Zheng, Zhong & Zhang, 2020). Consequently, demand for *A. argyi* continues to grow annually, and this has led to an expansion in its cultivation area (You et al., 2011). Given the history of the plant in traditional medicine, a standardized cultivation management plan will be necessary to support the scaling up of production to meet the growing global demand (Luo, 2021).

Plant spacing and density play crucial roles in agricultural crop yield and quality parameters (Ghanbari et al., 2016; Souri, 2016; Adnan et al., 2021; Shah et al., 2021). A reasonable planting density can improve the utilization efficiency of land, water and nutrients, thereby increasing the yield of medicinal materials (Farhadi, Souri & Palirezalu, 2013). Yang et al. (2009) demonstrated that appropriately increasing density can enhance the total biomass and leaf yield of *Artemisia annua* populations, as well as promote the formation of photosynthetic products and leaf yield. However, excessively high density significantly reduces artemisinin content. Chen (2011) found that the dry weight of individual plants and the biomass of each organ of *Prunella vulgaris* gradually decrease as planting density increases. Studies conducted by Zhao et al. (2013) and Zhang et al. (2007) have consistently demonstrated that excessively high planting densities can lead to reduced yields in medicinal plants. Planting density also significantly influences the content of active components in medicinal materials, with excessively dense planting sometimes lowering the levels of these components. This phenomenon can be attributed to intensified competition among plants, which leads to uneven nutrient distribution and impaired synthesis of secondary metabolites. Research by Gu et al. (2016) and Dou et al. (2009) has revealed that when planting density becomes too high, the content of active components in the medicinal parts of plants tends to decrease, resulting in diminished quality. This decline may be associated with insufficient root growth space in the soil, as with *Blumea balsamifera* under high-density conditions, which adversely affects its ability to absorb water and nutrients.

The growth and development stages of medicinal plants are closely related to the content of their active components. There are significant differences in the types and concentrations of chemical constituents in medicinal parts during different growth periods. Therefore, scientifically determining the harvesting time is a crucial factor in ensuring the quality of medicinal materials. Research indicates that the key active components in *A. argyi* leaves reach their peak levels when harvested around the Dragon Boat Festival. Chang et al. (2020) found that the total volatile oil content peaked around the Dragon Boat Festival (which fell on June 18 of that year), while Xue (2020) analysis further demonstrated that total flavonoids, total phenolic acids, and various volatile and non-volatile components also peak around this time. Zhang et al. (2016) detailed the changing pattern of volatile oil content, which continuously increases before the festival, peaks around May 20th, and gradually declines thereafter. These findings collectively provide scientific support for the traditional practice of harvesting *A. argyi* leaves around the Dragon Boat Festival, though a systematic analysis of the optimal harvesting time remains lacking. Tan et al. (2008) determined the active ingredient content for *Houttuynia cordata* harvested at different times in China and found that chlorogenic acid content was highest in spring while phenolic content was highest from May to July. Kang & Lee, (2011), Ozkan, Baydar & Erbas (2010) and Zheljazkov et al. (2010) studied *Perilla frutescens* leaves, *Origanum vulgare* leaves, and *Mentha haplocalyx* leaves in Korea, respectively, and found that each of the active ingredients and quality indexes differed significantly based on the harvest time of the herbs.

Planting density and harvesting time are important factors affecting the yield and quality of *A. argyi* leaves, but few studies have reported on the yield and quality of different planting densities of Spring-planted *A. argyi*. The best time for harvesting *A. argyi* leaves is generally thought to be immediately before and after the Dragon Boat Festival, but there is no study that examines the yield and quality of *A. argyi* leaves at different harvesting times in detail either. This study used *A. argyi* from Nanyang as the experimental material to analyze changes in agronomic traits and quality indicators of *A. argyi* at different planting densities and harvesting times, with the aim of optimizing both planting density and harvesting time in the standardized production of *A. argyi*.

## Materials & Methods

### Experiment material

The trial was conducted in 2023 at the moxa planting base of Henan Zhiyuantang Pharmaceutical Co., Ltd., Fangcheng County, Nanyang City, Henan Province (Latitude: 113.014702; Longitude: 33.366791; 218 m above sea level). The test material was Nanyang Ai No. 2 (Zhiyuan No. 1041).

### Experimental design

Uniform seedlings (with 6–7 leaves on the main stem) were separated from the mother plant and transplanted on March 29th. A randomized block design was adopted with four planting densities: D1 (20 cm × 20 cm), D2 (20 cm × 30 cm), D3 (20 cm × 40 cm), and D4 (20 cm × 50 cm), each with three replicates. Intra-row, the first dimension, spacing refers to the distance between adjacent plants within the same row, while inter-row spacing, the second dimension, refers to the distance between adjacent rows. Each plot covered an area of 20 m^2^, with 1-meter intervals between plots, and received conventional field management. Harvesting was conducted in the morning on June 6th, June 14th, June 22nd (Dragon Boat Festival), June 30th, and July 8th. During each harvest, 80 plants were randomly selected from each plot.

### Determination indexes and methods

#### Growth index measurements

Plant height was considered the vertical distance from the highest growing point of the plant to the ground. Crown width was defined as the maximum linear distance of plant leaves in the vertical direction. The number of effective leaves on the main stem was determined by counting all leaves on the main stem that were spread more than one-third. Dead leaf height was measured as the vertical distance from the highest dead leaf to the ground. The diameter of the middle stem was recorded, along with the average distance between the petioles of the four central leaves. The drying ratio was considered the ratio of the mass of dried *A. argyi* leaves to the mass of fresh *A. argyi* leaves. Measurements of plant height, crown width, and dead leaf height were obtained using a tape measure. Leaf spacing was measured with a straight ruler, and stem diameter was assessed using a Vernier caliper. Plant morphological indicators are the average of nine healthy plants. One m^2^ was harvested from each plot to measure yield.

### Quality index determination

#### Determination of moxa yield rate

After treatment, the stems and leaves of each sample were separated, and the leaves were dried in an oven at a constant temperature of 40 °C. From the treated sample leaves, 10 g of the dry product was extracted using the quartering method and placed into a high-speed universal crusher, rotating at 28,000 r min^−1^ for 30 s. The crushed mixture was then placed into a No. 2 sieve to sift the powder. The screened material was reintroduced into the mill and ground for another 30 s before being sieved again using the No. 2 sieve. The resulting moxa was weighed, and the moxa yield rate was calculated by dividing the mass of the moxa by the mass of the *A. argyi* leaves sample.

#### Determination of volatile oil content

The volatile oil content was determined with reference to the general rule 2204 “Determination of Volatile Oil” in the 2020 edition of the Chinese Pharmacopoeia (China Pharmacopoeia Committee, 2020).

#### Determination of index component content

The eucalyptol and borneol contents were determined by means of *A. argyi* leaves (Part 1) as described in the 2020 edition of the Chinese Pharmacopoeia (China Pharmacopoeia Committee, 2020).

#### Determination of total flavonoid, polysaccharide and polyphenol contents

To determine the total flavonoid, polysaccharide, and polyphenol contents, 0.5 g of moxa was carefully weighed and placed in a 40 mL conical flask. Subsequently, 20 mL of 70% ethanol was added, and the flask was weighed again to ensure the measurements were precise. The mixture underwent ultrasonic extraction at 60 °C for 1 h. Following the extraction, any weight loss was offset, and the solution was thoroughly shaken. A 10 mL aliquot of the solution was then transferred into a 10 mL centrifuge tube and centrifuged at 4,000 r/min for 5 min. The supernatant was carefully aspirated and diluted tenfold to obtain the test solution. Total flavonoid content was determined using the aluminum nitrate colorimetric method (Zhan et al., 2021), polysaccharide content was determined using the phenol-sulfuric acid method (Fang et al., 2021), and polyphenol content was determined using the ferrous tartrate colorimetric method (Deng & Han, 2020).

#### Determination of calorific value of moxa

A fully automatic calorimeter was used to measure the combustion calorfic value of moxa (Wu et al., 2020; Zou et al., 2023).

### Data processing and analysis

Microsoft Excel 2019 was used to organize the data, and the LSD method was used for significance testing. SPSS 19.0 (SPSS, Chicago, IL, USA) was used for correlation analysis and principal component analysis.

## Results

### Effect of different planting densities and harvesting dates on the growth, development and yield of *A. argyi*

It can be seen from Table 1 that across different harvesting dates, plant height, dead leaf height, leaf spacing and yield of *A. argyi* decreased as density decreased, while crown width, effective number of leaves on the main stem, stem diameter, and the drying ratio of *A. argyi* leaves increased as density decreased. Plant height, crown width, height of the tallest dead leaf, number of leaves on the main stem and yield of *A. argyi* leaves increased gradually as harvesting date was delayed. Likewise, stem diameter, leaf spacing and the drying ratio of *A. argyi* leaves first increased and then decreased as harvesting date was delayed. There was a significant (*P* < 0.05) difference in plant height between the lowest density (D1) and the other three densities, and these differences varied depending on the date of harvest. The plant heights across different densities of *A. argyi* all reached their maximum on July 8th and were significantly greater than the heights on June 6th, with the greatest increase at the D3 density (46.04%) and the smallest increase at D1 (29.60%). The dead leaf heights across different densities of *A. argyi* were significantly greater on July 8th than on June 6th, with D1, D2, D3, and D4 increasing in that month by 89.50%, 96.45%, 80.09%, and 65.29%, respectively. The number of effective leaves on the main stem was significantly greater at the D4 density than at the D1 density on all harvest dates except June 14th. The stem thickness was significantly greater at the D4 density than at the D1 density across all harvest dates. There was no significant difference in stem thickness between different densities on different harvesting dates. The drying ratio was significantly greater at the D4 density than at the D1 density across different harvesting dates, with D4 levels being 27.59%, 30.00%, 24.24%, 32.26%, and 23.33% greater than D1 levels on the harvest dates from June 6th to July 8th, respectively, and the maximum drying ratio of *A. argyi* leaves across different densities occurring on June 22nd. The yield was significantly smaller at the D4 density than at the D1 density across different harvesting dates. As harvesting date was further delayed, the increased *A. argyi* leaves yield gradually decreased across all densities, and the yield of all densities increased significantly, by 59.08%, 42.47%, 51.74%, and 48.29% from June 6th to July 8th, respectively. Proper thinning promoted increased stem thickness in *A. argyi*, compact leaf spacing, reduced rate of withered leaves, increased number of effective leaves, and increased the drying ratio of leaves. Dense planting can significantly increase *A. argyi* leaves yield, but it can also reduce the number of effective leaves on a single plant. Therefore, appropriately delayed harvesting can increase the number of effective leaves on the main stem as well as increase the yield of *A. argyi* leaves, but the rate of dead leaves would also be expected to increase.

**Table 1:** Effect of different planting densities and harvesting dates on the growth index and yield of *Artemisia argyi*. Lowercase letters indicate a significant difference between different densities on the same harvesting dates (*p* < 0.05), and uppercase letters indicate a significant difference between different harvesting dates at the same density (*p* < 0.05).

		Plant height/cm	Crown breadth/cm	Dead leaf height/cm	Number of effective leaves on main stem/slice	Stem diameter/mm	Blade pacing/cm	Drying ratio	Yield/ kg ha^−1^
June 6th	D1	118.22 ± 5.20Ca	28.40 ± 1.80Bb	22.29 ± 1.16Da	22.78 ± 2.54Cb	6.32 ± 0.50Ab	10.43 ± 0.77CDa	0.29 ± 0.03Ac	3007.65 ± 94.14Ca
	D2	103.34 ± 4.28Eb	35.82 ± 3.63Aa	17.48 ± 1.58Dab	26.78 ± 1.56Cab	6.99 ± 0.47Aab	9.23 ± 0.54ABb	0.32 ± 0.01Bb	2924.10 ± 78.89Cab
	D3	96.44 ± 4.03Ebc	37.30 ± 3.45Ba	15.77 ± 1.38Db	30.33 ± 2.00Da	7.27 ± 0.63Aab	8.01 ± 0.59Abc	0.34 ± 0.02Aab	2569.35 ± 96.10Dbc
	D4	90.98 ± 4.32Ec	39.67 ± 2.68Ba	15.30 ± 1.02Cb	30.78 ± 2.49Da	8.07 ± 0.63Aa	6.90 ± 0.27Ac	0.37 ± 0.02Ca	2381.70 ± 60.47Cc
June 14th	D1	134.44 ± 3.15Ba	30.59 ± 1.35ABb	28.66 ± 2.70Ca	27.67 ± 2.00BCa	6.42 ± 0.24Ac	10.67 ± 0.83BCa	0.30 ± 0.04Ac	3472.20 ± 125.55Ba
	D2	114.62 ± 8.34Db	36.94 ± 3.08Aab	20.52 ± 1.59CDb	32.44 ± 2.70Ba	7.04 ± 0.30Abc	9.73 ± 0.60Ab	0.33 ± 0.02ABb	3198.60 ± 47.46Cab
	D3	104.43 ± 3.11Dc	39.91 ± 1.71ABab	15.99 ± 1.38CDc	32.78 ± 1.99CDa	7.31 ± 0.20Ab	8.02 ± 0.63Ac	0.37 ± 0.02Aa	3005.70 ± 114.06Cab
	D4	100.12 ± 6.03Dc	43.11 ± 2.06ABa	16.23 ± 1.50Cc	32.11 ± 1.62Da	8.04 ± 0.64Aa	7.48 ± 0.88Ac	0.39 ± 0.02ABCa	2811.90 ± 94.90ABCb
June 22nd	D1	140.03 ± 5.37Ba	31.91 ± 1.85ABc	34.73 ± 2.57Ba	29.56 ± 2.83ABCb	6.69 ± 0.32Ab	11.12 ± 0.81Aa	0.33 ± 0.04Ac	4451.70 ± 71.58Aa
	D2	122.51 ± 6.49Cb	38.17 ± 2.40Ab	26.06 ± 2.12BCb	34.89 ± 2.09Bab	7.07 ± 0.32Ab	9.76 ± 0.72Ab	0.36 ± 0.02Abc	3762.60 ± 161.45Bb
	D3	114.19 ± 3.79Cc	41.98 ± 3.13Aab	21.92 ± 2.76BCb	36.22 ± 2.22BCa	7.86 ± 0.28Aa	8.14 ± 0.58Abc	0.38 ± 0.03Aab	3510.00 ± 63.11Bbc
	D4	111.89 ± 3.04Cc	44.76 ± 2.38ABa	20.98 ± 2.26Bb	37.44 ± 2.70Ca	8.18 ± 0.13Aa	7.26 ± 0.42Ac	0.41 ± 0.02Aa	3132.75 ± 107.32ABc
June 30th	D1	148.07 ± 4.25Aa	32.22 ± 1.86Bc	39.72 ± 3.04ABa	33.00 ± 2.78ABb	6.80 ± 0.26Ab	10.90 ± 0.83ABa	0.31 ± 0.03Ab	4734.75 ± 119.90Aa
	D2	131.42 ± 3.62Bb	38.56 ± 3.60Ab	31.53 ± 2.78ABb	36.33 ± 2.60Bab	7.12 ± 0.52Ab	9.80 ± 0.55Aa	0.32 ± 0.02Bb	3983.85 ± 44.79ABb
	D3	127.83 ± 4.46Bb	43.56 ± 2.86Aa	25.73 ± 1.31ABbc	38.33 ± 2.29ABa	7.72 ± 0.47Aab	8.61 ± 0.72Ab	0.38 ± 0.04Aa	3839.40 ± 146.94ABb
	D4	124.12 ± 3.47Bb	45.66 ± 1.56ABa	22.97 ± 1.65ABc	39.67 ± 2.00Ba	8.34 ± 0.50Aa	7.48 ± 0.36Ac	0.41 ± 0.04ABa	3457.65 ± 195.76Ab
July 8th	D1	153.21 ± 2.83Aa	33.91 ± 1.29Ac	42.24 ± 2.18Aa	35.11 ± 2.37Ab	6.34 ± 0.25Ac	9.71 ± 0.66Da	0.30 ± 0.01Ac	4784.85 ± 144.05Aa
	D2	143.37 ± 4.81Ab	39.85 ± 2.14Ab	34.34 ± 1.83Ab	40.22 ± 3.19Aa	6.96 ± 0.22Ab	8.87 ± 0.32Bab	0.32 ± 0.02Bbc	4165.95 ± 162.84Aab
	D3	140.84 ± 3.57Ab	44.89 ± 2.41ab	28.40 ± 2.06Abc	42.44 ± 2.51Aa	7.64 ± 0.29Aa	8.22 ± 0.49Abc	0.35 ± 0.03Aab	3898.95 ± 149.55Ab
	D4	131.88 ± 6.19Ac	47.32 ± 2.64Aa	25.29 ± 2.33Ac	43.22 ± 1.99Aa	7.93 ± 0.30Aa	7.39 ± 0.43Ac	0.37 ± 0.03BCa	3531.60 ± 87.39Ab

### Effect of different planting densities and harvesting dates on quality of *A. argyi*

#### Effect of different planting densities and harvesting dates on moxa yield rate of *A. argyi* leaves

**Figure 1:**
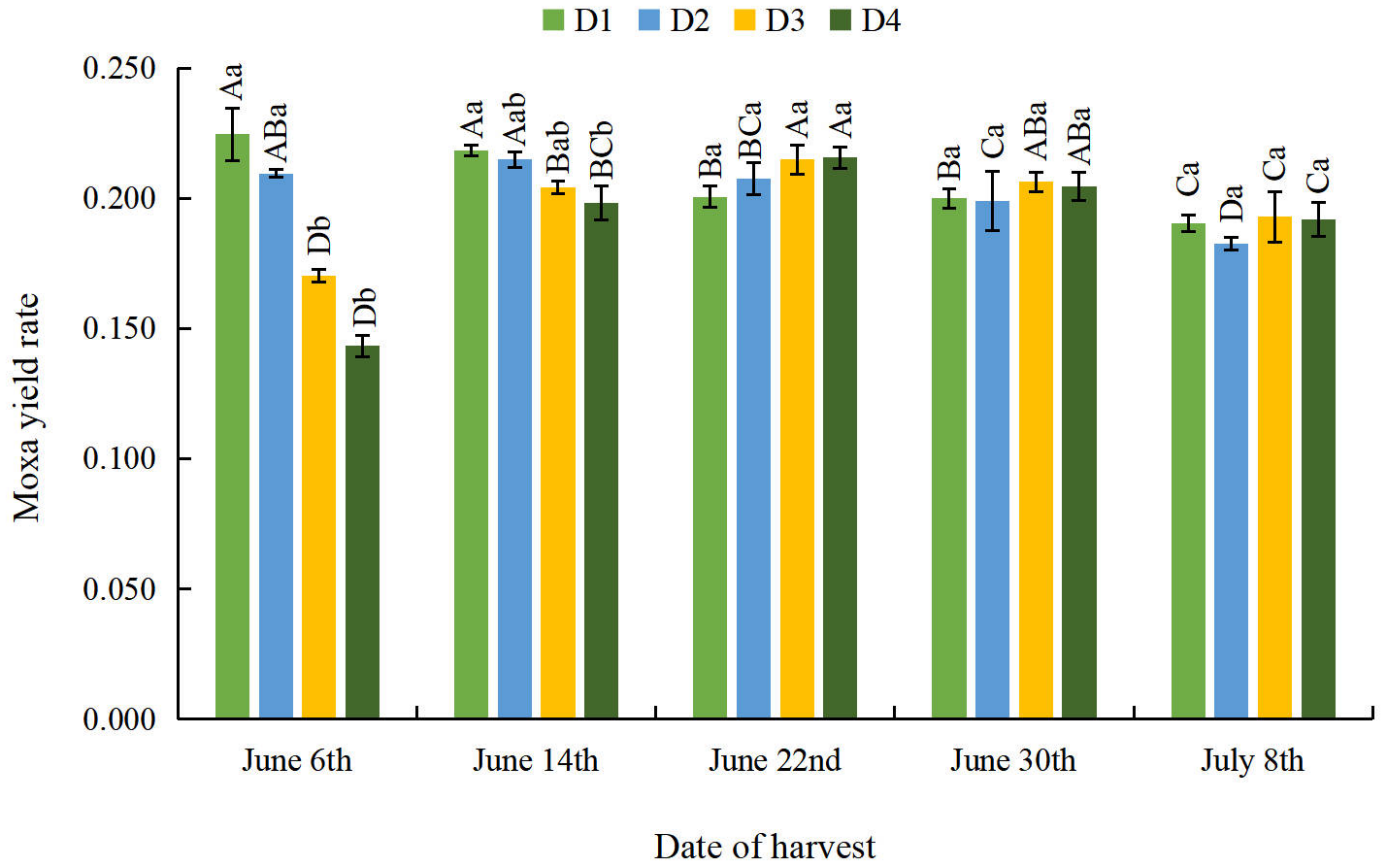
Effect of different planting densities and harvesting dates on the moxa yield rate of *Artemisia argyi* leaves. Lowercase letters indicate a significant difference between different densities on the same harvesting dates (*p* < 0.05), and uppercase letters indicate a significant difference between different harvesting dates at the same density (*p* < 0.05), the same below.

As can be seen from Fig. 1, the moxa yield rate of *A. argyi* leaves gradually decreased on June 6th and June 14th as density decreased. There was no significant difference in the moxa yield rate of *A. argyi* leaves on the other three harvesting dates. With the delay of the harvesting date, the moxa yield rate gradually decreased at the D1 density, and the moxa yield rate showed a trend of initial increase and subsequent decrease at the other three densities. At the D2 density, the moxa yield rate reached its maximum value on June 14th, and both the D3 and D4 densities reached their maximum values on June 22nd. This demonstrates that appropriate sparse planting can improve the moxa yield rate of *A. argyi* leaves.

#### Effect of different planting densities and harvesting dates on volatile oil content in *A. argyi* leaves

**Figure 2:**
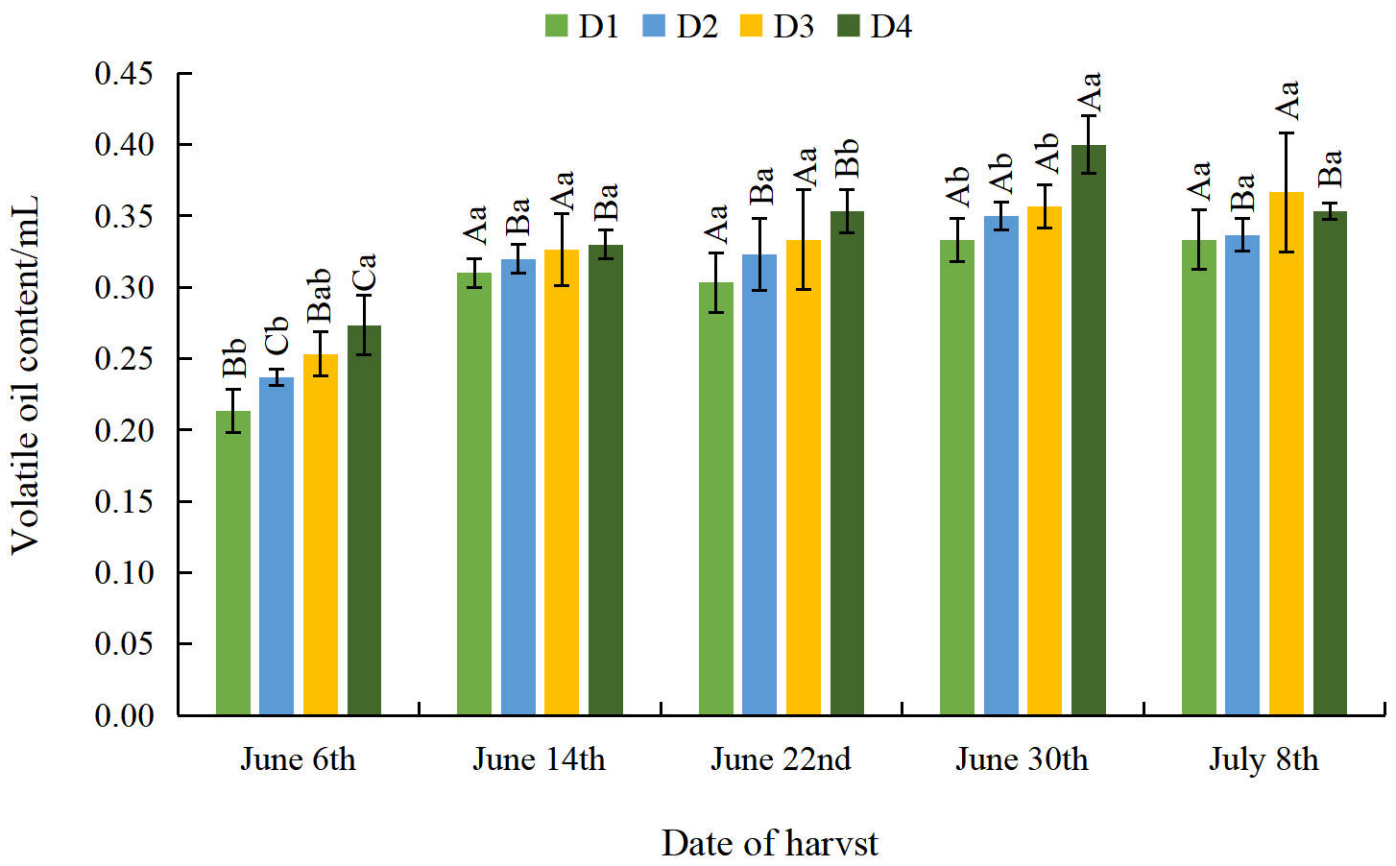
Effect of different planting densities and harvesting dates on volatile oil content of *A. argyi* leaves. The volatile oil content was obtained from 40 g of *A. argyi* leaves.

As the harvesting dates progressed, the volatile oil content of *A. argyi* leaves gradually increased as density decreased (Fig. 2). The volatile oil content of *A. argyi* leaves was significantly greater at the D4 density than at the D1 density on June 6th, June 22nd, June 30th and October 14th (Table S1). This indicates that the volatile oil content of *A. argyi* leaves can be significantly augmented by appropriately reducing the planting density. With the delay of harvest date, the overall volatile oil content of *A. argyi* leaves first increased and then slightly decreased across all densities. The volatile oil content of *A. argyi* leaves reached its minimum value on June 6th across all density treatments, and reached its maximum value on June 30th at the D1, D2, and D4 densities. At the D3 density, the volatile oil content of *A. argyi* leaves reached its maximum value on July 8th. Therefore, appropriately delaying harvesting can significantly increase the volatile oil content of *A. argyi* leaves.

#### Effect of different planting densities and harvesting dates on eucalyptol content in *A. argyi* leaves

**Figure 3:**
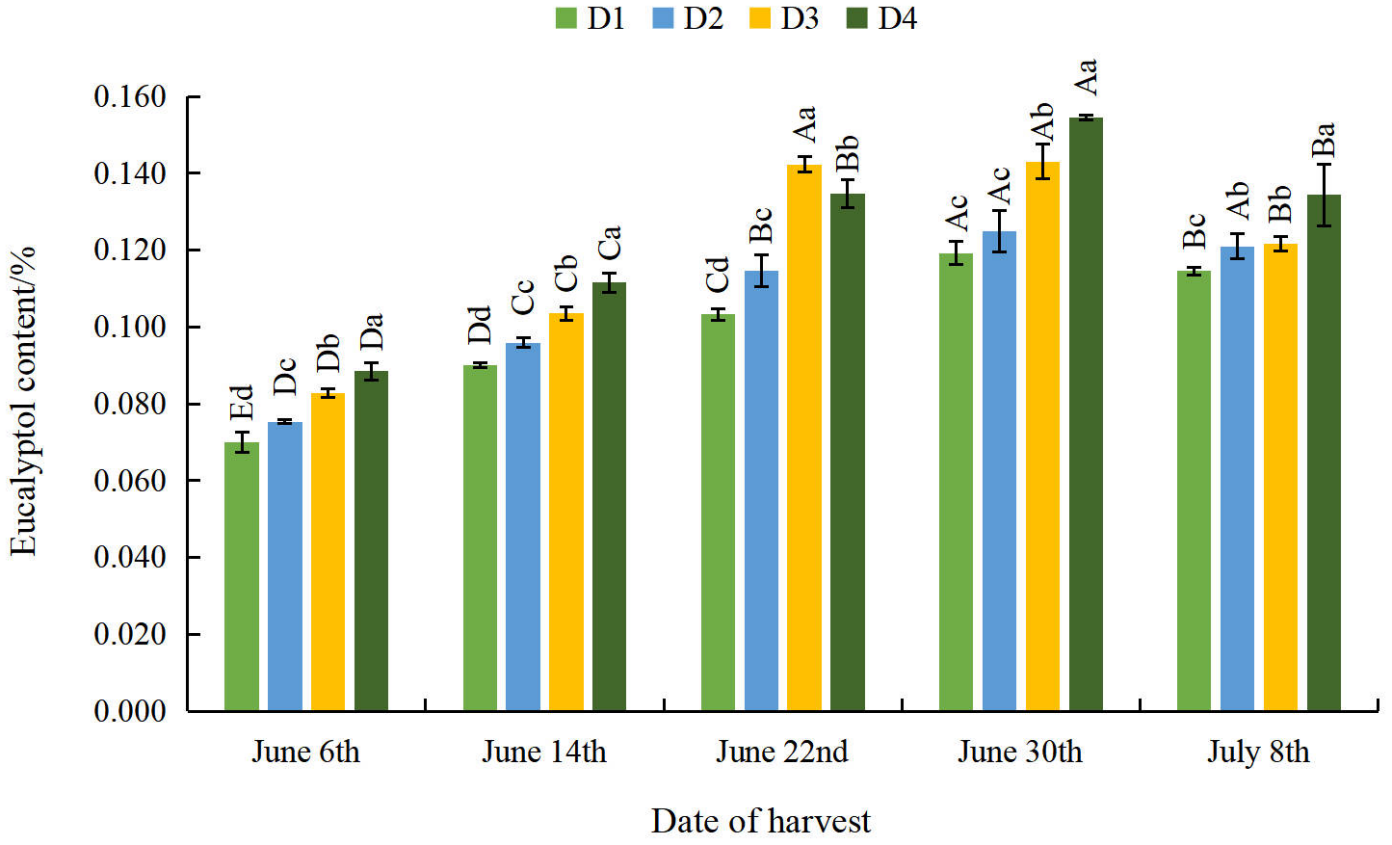
Effect of different planting densities and harvesting dates on eucalyptol content in *Artemisia argyi* leaves.

The eucalyptol content of *A. argyi* leaves increased gradually as density decreased, even as harvest was further delayed (Fig. 3). The eucalyptus oleoresin content of *A. argyi* leaves was significantly greater at the D4 density than at the D1 density. This indicates that the eucalyptol content of *A. argyi* leaves can be increased by enacting appropriate sparse planting. With the delay of the harvesting date, the eucalyptol content of *A. argyi* leaves in each density treatment showed a trend of initial increase and subsequent decrease. The eucalyptol content reached its minimum on June 6th across all densities, and the eucalyptol content reached its maximum on June 30th at the D1, D2 and D4 densities. At the D3 density, the eucalyptol content reached its maximum on June 22nd. These results indicate that delaying the harvest can significantly increase the eucalyptol content of *A. argyi* leaves.

#### Effect of different planting densities and harvesting dates on borneol content of *A. argyi* leaves

**Figure 4:**
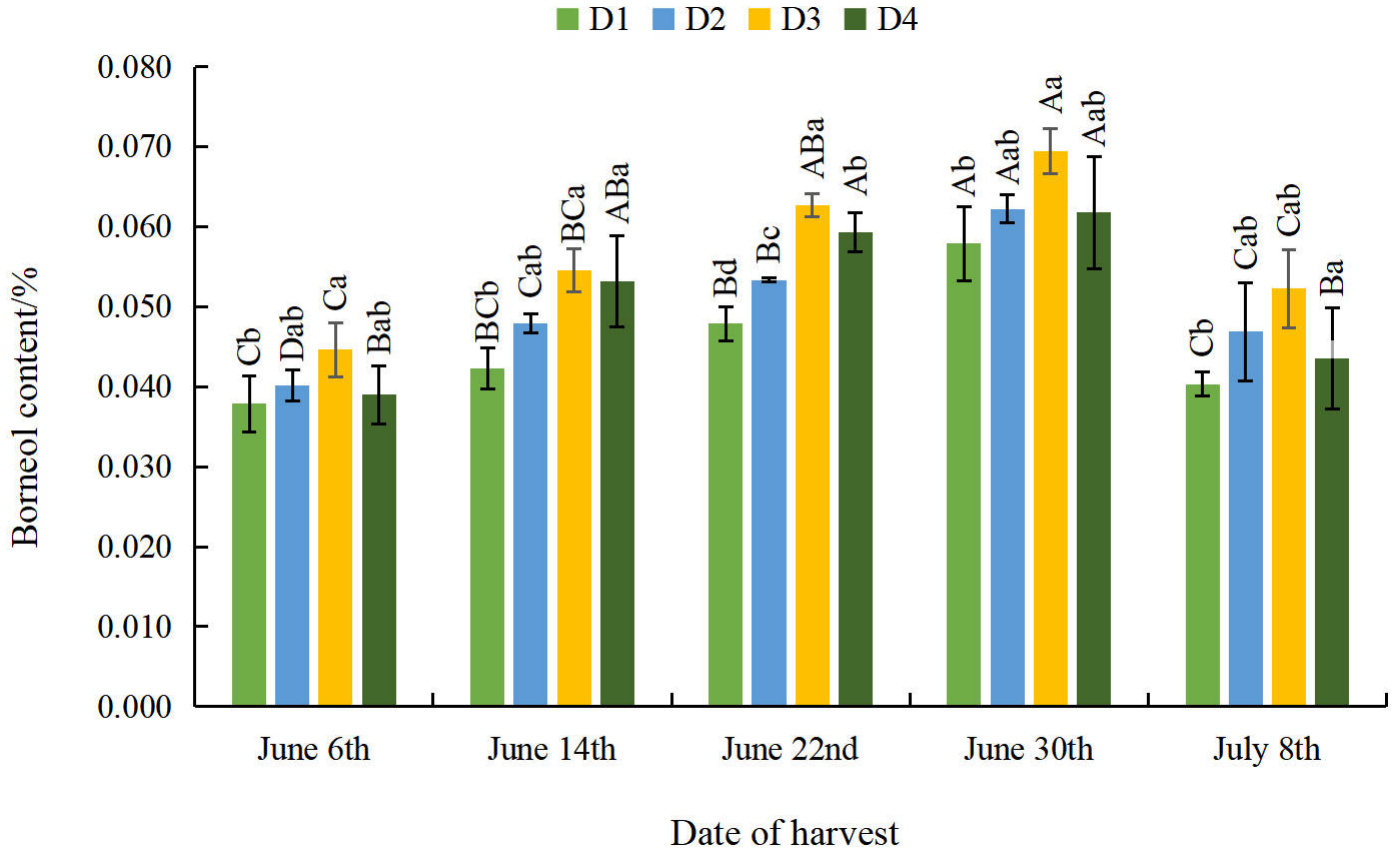
Effect of different planting densities and harvesting dates on borneol content of *Artemisia argyi* leaves.

Regardless of delay to harvest, the borneol content of *A. argyi* leaves increased at first and then decreased as density decreased (Fig. 4). The maximum value was observed at the D3 density at a significantly higher level than at the D1 density across all harvesting dates, which indicates that appropriate sparse planting can significantly increase the borneol content of *A. argyi* leaves. As the harvesting date was delayed, the borneol content of *A. argyi* leaves showed a trend of initial increase and subsequent decrease across all densities. The borneol content of *A. argyi* leaves reached its minimum value on June 6th and its maximum value on June 30th. This shows that appropriately delaying harvest can significantly increase the borneol content of *A. argyi* leaves.

#### Effect of different planting densities and harvesting dates on combustion calorific value of moxa

**Figure 5:**
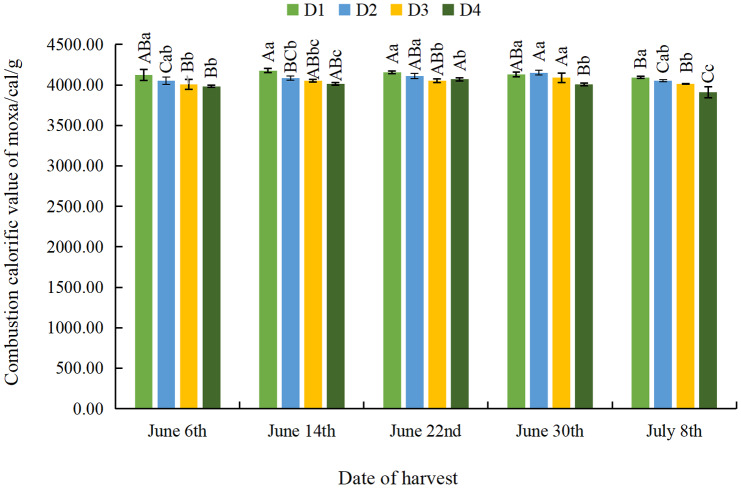
Effect of different planting densities and harvesting dates on combustion calorific value of moxa.

As estimated by calorimetry, the combustion calorific value of moxa gradually decreased as density decreased regardless of delay to harvest (Fig. 5). The combustion calorific value of moxa was significantly smaller at the D4 density than at the D1 density, which indicates that appropriately sparse planting could significantly increase the combustion calorific value of moxa. With the delay of the harvesting time, the combustion calorific value of moxa showed a trend of initial increase and subsequent decrease across all density treatments, reaching its highest level on June 14th at 4,178.33 cal/g for the D1 density; on June 30th at 4,150.33 and 4,089.00 cal/g for the D2 and D3 densities, respectively; and on June 22nd at 4,068.67cal/g for the D4 density.

#### Effect of different planting densities and harvesting dates on total flavonoid content of moxa

**Figure 6:**
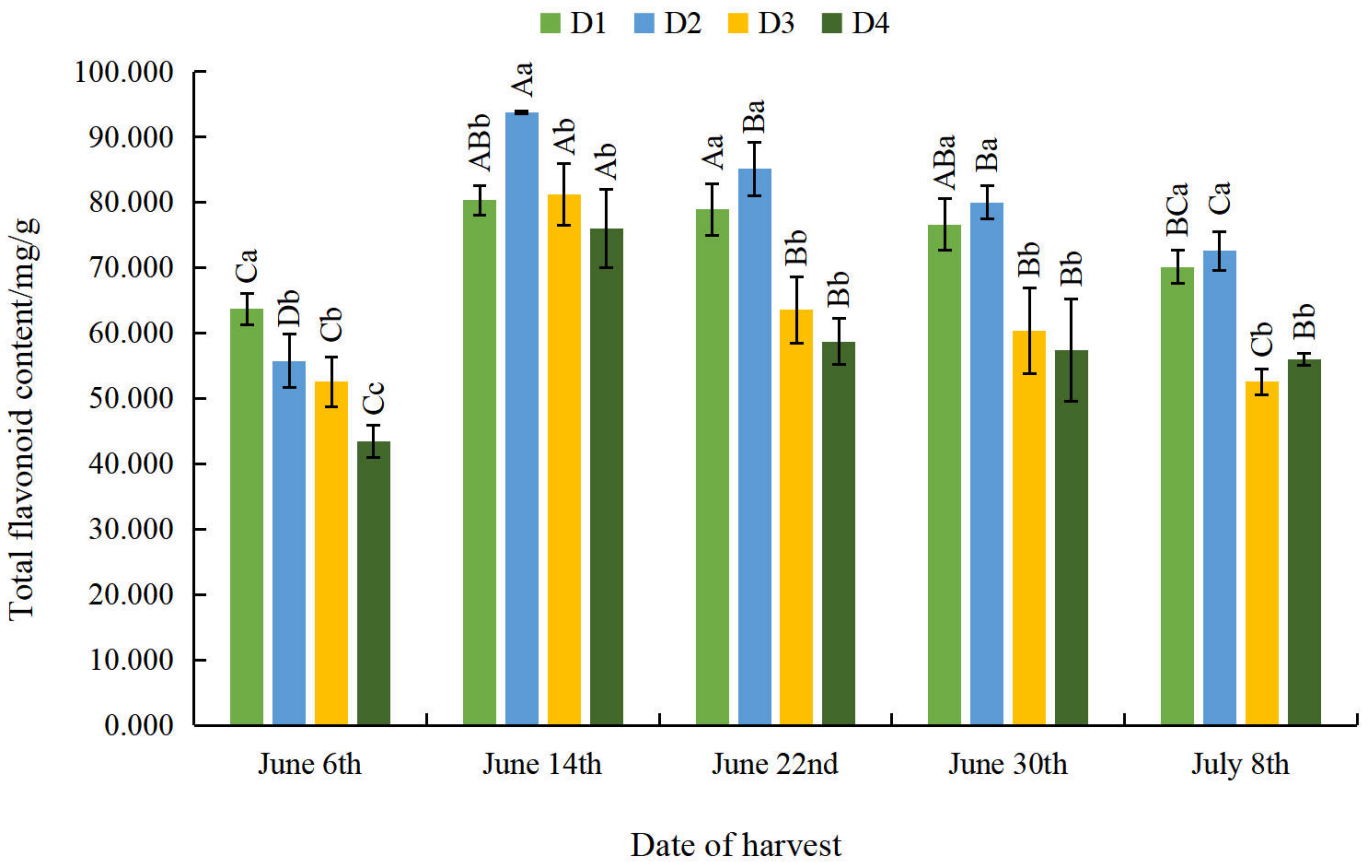
Effect of different planting densities and harvesting dates on total flavonoid content of moxa.

The total flavonoid content of moxa initially increased and subsequently decreased as density decreased across all harvesting dates, except for June 6th (Fig. 6). From June 14th to July 8th, all flavone contents reached their maximum value at the D2 density, thus indicating that appropriate sparse planting can significantly increase the total flavonoid content of moxa. With the delay of the harvesting date, the total flavonoid contents followed a trend of initial increase and subsequent decrease across all densities, reaching their maximum values on June 14th, at levels 26.10%, 68.20%, 54.73% and 74.84% higher than the minimum values across the four density treatments, respectively. This indicates that appropriate early harvesting can significantly increase the total flavonoid content of moxa.

**Figure 7:**
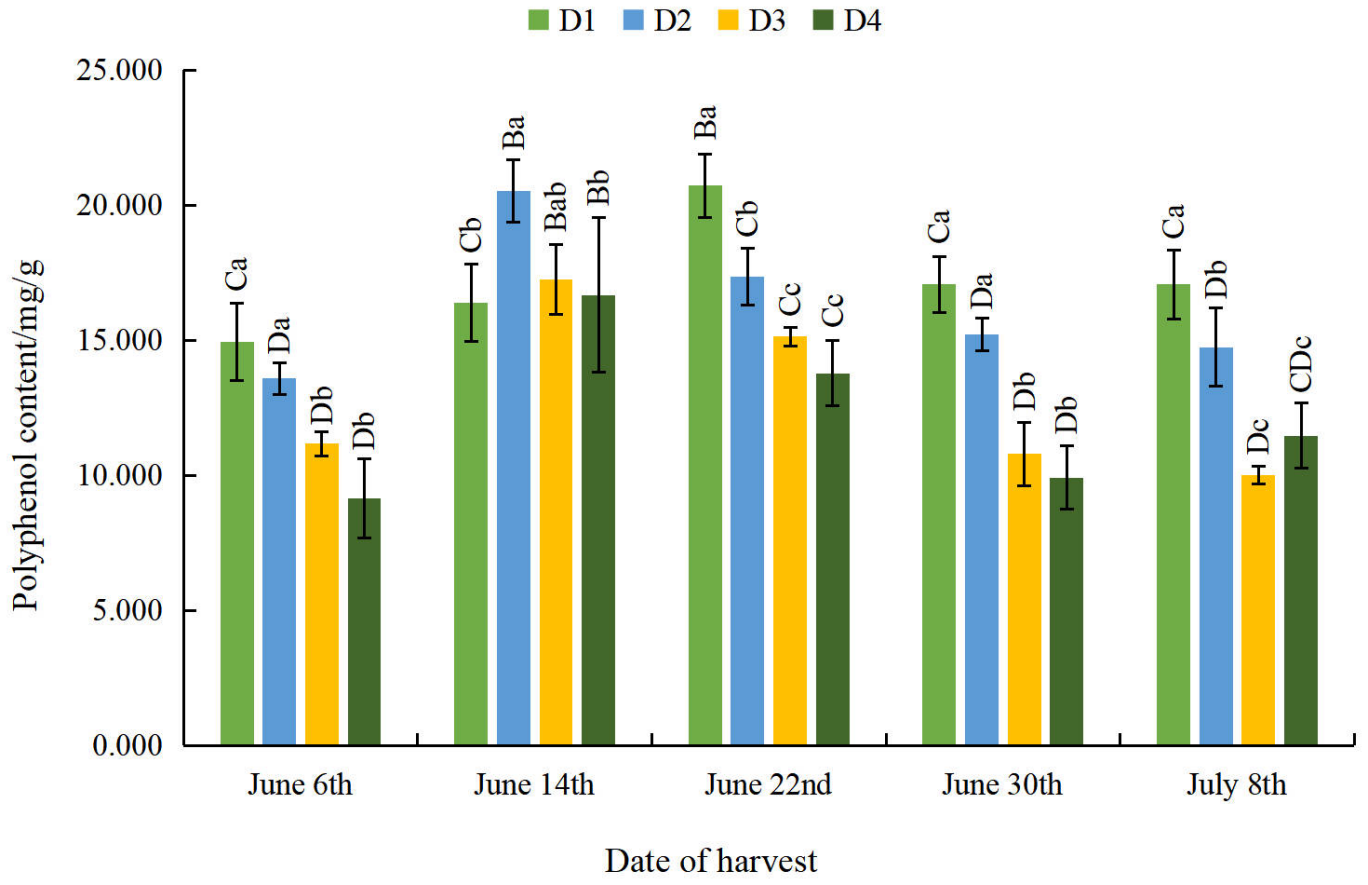
Effect of different planting densities and harvesting dates on polyphenol content of moxa.

#### Effect of different planting densities and harvesting dates on polyphenol content of moxa

The overall polyphenol content of moxa decreased as density decreased across all harvesting dates except for June 14th (Fig. 7). The polyphenol content of moxa decreased to its lowest at the D4 density on June 6th, June 22nd and June 30th. These levels differed significantly from those of the D1 density. The polyphenol content of moxa decreased to its lowest at the D3 density on July 8th. This level differed significantly than that of the D1 density and was 41.31% lower, which indicates that appropriate sparse planting could significantly increase the polyphenol content of moxa. With the delay of the harvesting date, the polyphenol content showed a trend of initial increase and subsequent decrease across all densities, with the polyphenol content of the D1 density reaching its maximum on June 22nd. The other three densities reached their maximum polyphenol contents on June 14th, indicating that appropriate early harvesting can significantly increase the polyphenol content of the moxa.

#### Effect of different planting densities and harvesting dates on polysaccharide content of moxa

**Figure 8:**
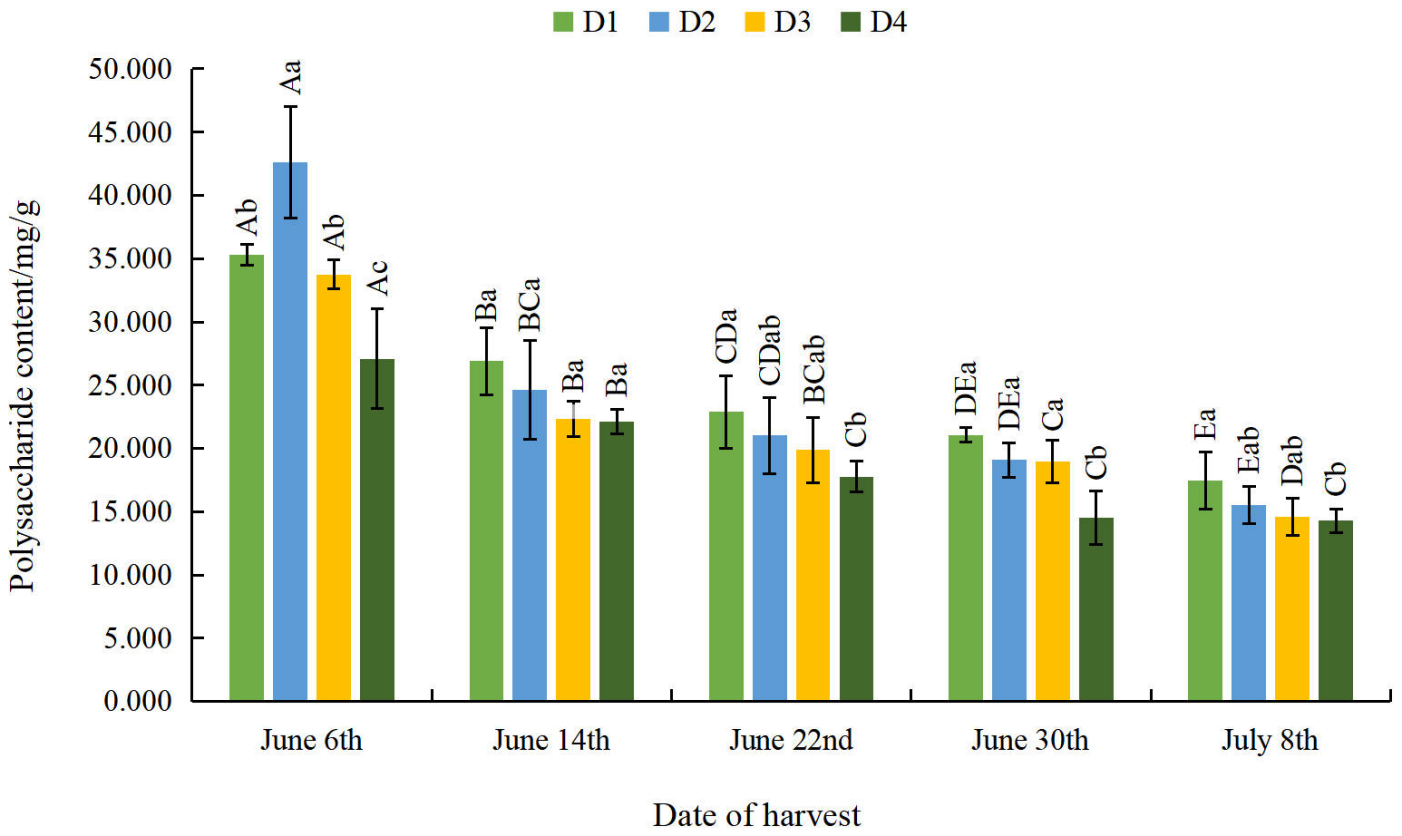
Effect of different planting densities and harvesting dates on polysaccharide content in moxa.

The moxa polysaccharide content gradually decreased as density decreased and as the harvesting date was delayed, but not on June 6th (Fig. 8). On June 14th, the difference in moxa polysaccharide content among the density treatments was not obvious. From June 22nd to July 8th, the moxa polysaccharide content was significantly lower at the D4 density than at the D1 density. As the harvest date was further delayed, the moxa polysaccharide content gradually decreased at all densities. The moxa polysaccharide content was significantly lower on July 8th than on June 6th, by 50.46%, 63.56%, 56.71%, and 47.30% at the four densities, respectively. This shows that both appropriate sparse planting and appropriate early harvesting can augment the moxa polysaccharide content.

### Principal component analysis of quality indexes of *A. argyi* at different planting densities and harvesting dates

Evaluating the quality of *A. argyi* requires the review of a combination of different indicators. The principal component analysis method can transform multiple interrelated indicators into a few unrelated comprehensive indicators without losing any original information. On this basis, membership function analysis was used. This method calculates a reliable and comprehensive score for each density, thus allowing for easy comparison (Tian et al., 2015; Liu et al., 2023). The volatile oil content, the moxa yield rate, calorific value of combustion, total flavonoid content, polysaccharide content and polyphenol content, eucalyptol content, borneol content, drying ratio and yield of *A. argyi* were analyzed at different planting densities and harvesting dates using principal component analysis and subordinate function analysis, and then the suitable harvesting date of *A. argyi* could be determined.

**Figure 9:**
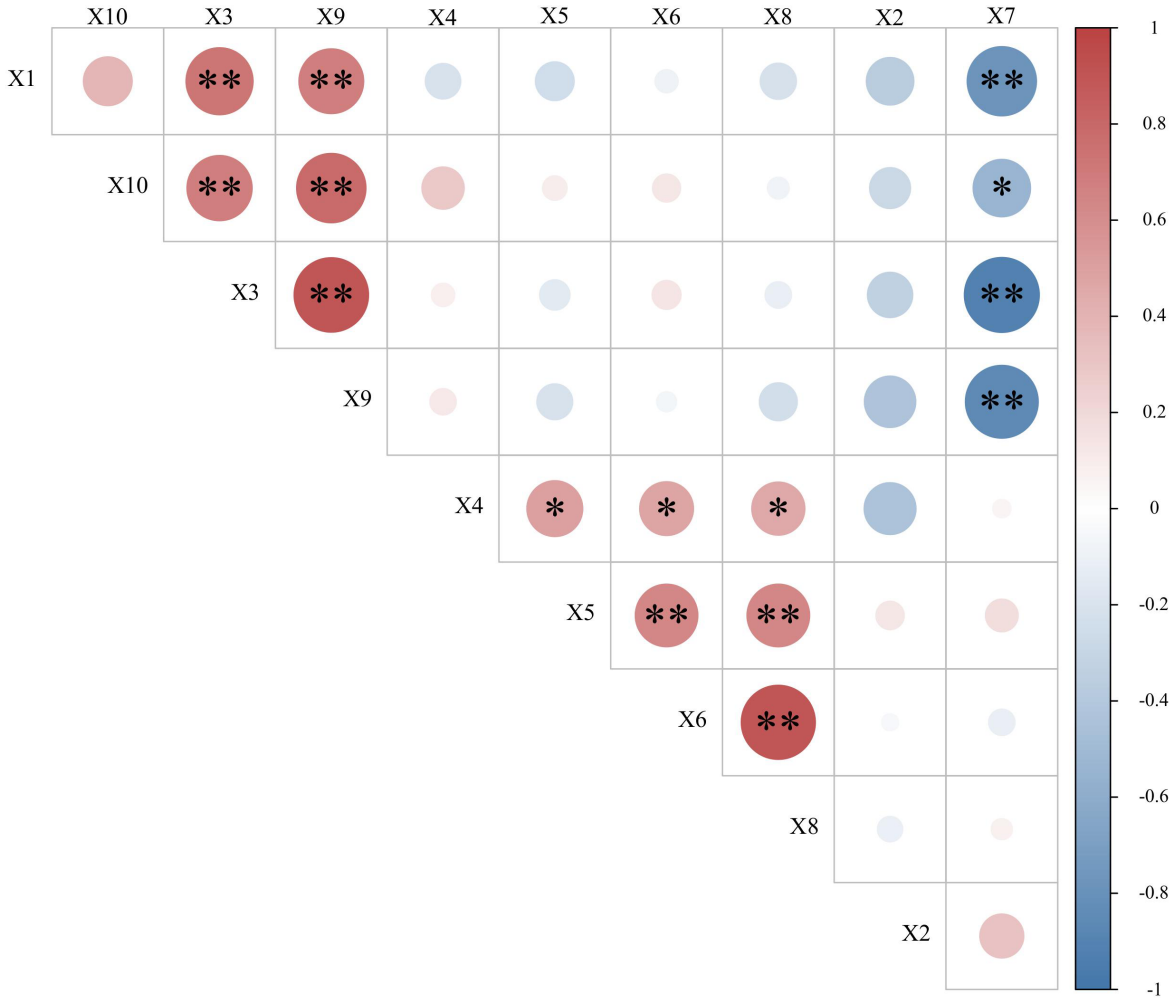
Correlations among key indicators of *artemisia argyi* across planting densities and harvesting dates. X1 (Drying ratio), X2 (Yield), X3 (Volatile oil content), X4 (Moxa yield rate), X5 (Combustion calorific value of moxa), X6 (Total flavonoid content), X7 (Polysaccharide content), X8 (Polyphenol content), X9 (Eucalyptol content), X10 (Borneol content). Correlations marked with ** are significant at *p*  <  0.01, and those marked with * are significant at *p*  <  0.05.

As shown in Fig. 9, under different planting densities and harvesting dates, the volatile oil content in *A. argyi* leaves exhibited highly significant positive correlations with both the drying ratio and borneol content. Eucalyptol content also demonstrated highly significant positive correlations with the drying ratio, borneol content, and volatile oil content. In contrast, polysaccharide content showed highly significant negative correlations with the drying ratio, volatile oil content, and eucalyptol content, along with a significant negative correlation with borneol content. The moxa yield rate was significantly positively correlated with both the calorific value and total flavonoid content, as well as polyphenol content. The calorific value showed highly significant positive correlations with total flavonoid content and polyphenol content, and a highly significant positive correlation was also observed between total flavonoid content and polyphenol content. However, no significant correlations were observed between yield and any of the quality indicators examined.

Principal component analysis was performed on the 10 quality-related indexes for different harvesting dates of *A. argyi* at the D1 density (Table 2), according to the principle that the eigenvalues of principal component factors are greater than 1 and the cumulative contribution rate exceeds 85%. The 10 indexes were downscaled to two principal components with a cumulative contribution rate of 85.013%, and the contribution rate of the first principal component was 63.350%, with larger loadings in the indexes of volatile oil content, the moxa yield rate, polysaccharide content, eucalyptol content, drying ratio, yield, etc. The second principal component had a contribution of 21.663%, with large loadings only in the index of combustion calorific value. The coefficients of the principal components were obtained by dividing the load vector in the principal component load matrix by the arithmetic square root of the characteristic root of each principal component. Then the values of the composite indexes (D value) on different harvesting dates were found based on the coefficients of the principal components and the values of each individual index. Under the D1 density, the D value was largest treatment on June 30th (0.797), followed by the value on June 22nd. The D value was the smallest on June 6th (Table 3). This indicates that the D1 density had the best comprehensive performance on June 30th, making it the most suitable date for harvesting, while June 22nd was the second most suitable date.

**Table 2:** Principal component analysis of quality related indexes of *Artemisia argyi* at different harvesting dates.

Index	First principal component	Second principal component
Volatile oil content	0.889	0.021
Moxa yield rate	−0.903	0.400
Calorific value of combustion	−0.124	0.974
Total flavone content	0.606	0.739
Polysaccharide content	−0.939	0.244
Polyphenol content	0.715	0.395
Eucalyptol content	0.955	−0.219
Borneol content	0.665	0.268
Drying ratio	0.825	0.314
Yield	0.959	−0.277
Eigenvalue	6.335	2.166
Contribution rate /%	63.350	21.663
Cumulative contribution rate /%	63.350	85.013

**Table 3:** μ (x), D values and comprehensive evaluation of different harvesting dates under the D1 density. H1 (June 6 th ), H2 (June 14 th ), H3 (June 22 nd ), H4 (June 30 th ), H5 (July 8 th ), D1 (20 cm × 20 cm), D2 (20 cm × 30 cm), D3 (20 cm × 40 cm), D4 (20 cm × 50 cm). This applies for Tables 5, 7 and 9 also.

Treatment	μ(1)	μ(2)	D-value	Synthetic sort
H1D1	0.000	0.530	0.135	5
H2D1	0.300	1.000	0.479	4
H3D1	0.814	0.638	0.769	2
H4D1	0.949	0.355	0.797	1
H5D1	1.000	0.000	0.745	3

Principal component analysis was performed on 10 quality-related indicators of *A. argyi* at different harvest dates under the D2 density. The 10 indicators were dimensionally reduced into three principal components, with a cumulative contribution rate of 92.821% (Table 4). The first principal component contribution rate was 55.301%, with relatively large loads in volatile oil content, polysaccharide content, eucalyptol content, borneol content, yield and other indicators. The second principal component contribution rate was 26.775%, with relatively large loads in indicators such as the moxa yield rate and polyphenol content. Under the D2 density, the D value was the largest on June 30th (0.918), followed by the value on June 22nd. The D value was the smallest on June 6th (Table 5). This indicates that June 30th was the most suitable harvesting date for the D2 density, followed by June 22nd.

**Table 4:** Principal component analysis of quality-related indexes of *Artemisia argyi* at different harvesting dates under the D2 density.

Index	First principal component	Second principal component	Third principal component
Volatile oil content	0.973	−0.038	−0.186
Moxa yield rate	−0.442	0.868	0.201
Calorific value of combustion	0.699	0.328	0.602
Total flavone content	0.662	0.676	−0.312
Polysaccharide content	−0.951	0.110	0.280
Polyphenol content	0.262	0.840	−0.464
Eucalyptol content	0.967	−0.234	0.031
Borneol content	0.861	0.132	0.443
Drying ratio	0.245	0.592	0.217
Yield	0.867	−0.466	−0.024
Eigenvalue	5.530	2.677	1.075
Contribution rate /%	55.301	26.775	10.746
Cumulative contribution rate /%	55.301	82.075	92.821

**Table 5:** μ (x), D values and comprehensive evaluation of different harvesting dates under the D2 density.

Treatment	μ(1)	μ(2)	μ (3)	D-value	Synthetic sort
H1D2	0.000	0.474	0.242	0.165	5
H2D2	0.487	1.000	0.219	0.604	3
H3D2	0.779	0.715	0.565	0.736	2
H4D2	1.000	0.716	1.000	0.918	1
H5D2	0.658	0.000	0.000	0.392	4

Principal component analysis was performed on 10 quality-related indicators of *A. argyi* at different harvest dates under the D3 density. The 10 indicators were dimensionally reduced into two principal components, with a cumulative contribution rate of 87.626% (Table 6). The contribution rate of the first principal component was 61.343%, with large loadings on volatile oil content, moxa yield rate, combustion calorific value, polysaccharide content, eucalyptol content, borneol content and drying ratio. The contribution rate of the second principal component was 26.283%, with large loadings on indicators such as total flavonoid content and polyphenol content. Under the D3 density, the D value was the largest on June 30th (0.821), followed by the value on June 14th. The smallest D value was on June 6th (Table 7). This indicates that June 30th was the most suitable harvest time for the D3 density, followed by June 14th.

**Table 6:** Principal component analysis of quality related indexes of *Artemisia argyi* at different harvesting dates under the D3 density.

Index	First principal component	Second principal component
Volatile oil content	0.828	−0.382
Moxa yield rate	0.950	0.240
Calorific value of combustion	0.811	0.279
Total flavone content	0.373	0.840
Polysaccharide content	−0.801	0.408
Polyphenol content	0.242	0.906
Eucalyptol content	0.918	−0.283
Borneol content	0.927	0.018
Drying ratio	0.864	0.438
Yield	0.777	−0.618
Eigenvalue	6.134	2.628
Contribution rate/%	61.343	26.283
Cumulative contribution rate/%	61.343	87.626

**Table 7:** μ (x), D values and comprehensive evaluation of different harvesting dates under the D3 density.

Treatment	μ (1)	μ (2)	D-value	Synthetic sort
H1D3	0.000	0.762	0.228	5
H2D3	0.549	1.000	0.684	2
H3D3	0.691	0.529	0.642	3
H4D3	1.000	0.403	0.821	1
H5D3	0.596	0.000	0.417	4

Principal component analysis was performed on 10 quality-related indicators of *A. argyi* at different harvest dates under the D4 density. The 10 indicators were dimensionally reduced into three principal components, with a cumulative contribution rate of 98.095% (Table 8). The contribution rate of the first principal component was 60.590%, with large loadings on volatile oil content, moxa yield rate, polysaccharide content, eucalyptol content, borneol content and drying ratio. The contribution rate of the second principal component was 25.121%, with a large loading on the combustion calorific value. Under the D4 density, the D value was the largest on June 22nd (1.000), followed by June 30th (Table 9). The D value was the smallest on June 6th. This indicates that June 22nd was the most suitable harvest date for the D4 density, followed by June 30th.

**Table 8:** Principal component analysis of quality related indexes of *Artemisia argyi* at different harvesting dates under the D4 density.

Index	First principal component	Second principal component	Third principal component
Volatile oil content	0.936	−0.298	0.029
Moxa yield rate	0.969	0.146	−0.114
Calorific value of combustion	0.363	0.745	0.552
Total flavone content	0.543	0.562	−0.588
Polysaccharide content	−0.823	0.539	0.136
Polyphenol content	0.357	0.743	−0.548
Eucalyptol content	0.917	−0.387	0.092
Borneol content	0.905	0.249	0.299
Drying ratio	0.854	0.380	0.351
Yield	0.790	−0.576	−0.185
Eigenvalue	6.059	2.512	1.238
Contribution rate /%	60.590	25.121	12.383
Cumulative contribution rate /%	60.590	85.712	98.095

**Table 9:** μ (x), D values and comprehensive evaluation of different harvesting dates under the D4 density.

Treatment	μ(1)	μ(2)	μ (3)	D-value	Synthetic sort
H1D4	0.000	0.689	0.710	0.266	5
H2D4	0.660	0.892	0.577	0.709	3
H3D4	1.000	1.000	1.000	1.000	1
H4D4	0.940	0.529	0.593	0.791	2
H5D4	0.597	0.000	0.000	0.369	4

### Comprehensive evaluation of *A. argyi* leaves quality at different planting densities on the most suitable harvesting dates

The quality indexes of *A. argyi* leaves were analyzed at different densities on the most suitable harvesting dates for each density. Table 10 shows that volatile oil contents and moxa yield rates did not differ significantly among densities. Meanwhile, eucalyptol content and borneol content reached their maximum values at the D3 density on June 30th, the drying ratio reached its maximum value at the D4 density, and the yield reached its maximum value at the D1 density. Analysis of the comprehensive quality indexes and yield of *A. argyi* found that a planting density of 20 cm × 40 cm and a harvesting date of June 30th were the most suitable for optimizing both quality and yield.

**Table 10:** Comparison of quality of different densities at the most suitable harvesting date. Lowercase letters indicate a significant difference in the optimum harvesting period for different densities (*p* < 0.05).

Treatment	Date of harvest	Volatile oil content /mL	Moxa yield rate	Eucalyptol content/%	Borneol content/%	Drying ratio	Per mu yield/kg
D1	June 30th	0.33 ± 0.02a	0.20 ± 0.004a	0.12 ± 0.003b	0.06 ± 0.005b	0.31 ± 0.03b	315.65 ± 17.64a
D2	June 30th	0.35 ± 0.01a	0.20 ± 0.011a	0.12 ± 0.005b	0.06 ± 0.002b	0.32 ± 0.02b	265.59 ± 16.43b
D3	June 30th	0.36 ± 0.02a	0.21 ± 0.004a	0.14 ± 0.005a	0.07 ± 0.003a	0.38 ± 0.04ab	255.96 ± 10.08b
D4	June 22nd	0.35 ± 0.02a	0.22 ± 0.004a	0.13 ± 0.004ab	0.06 ± 0.002b	0.41 ± 0.02a	208.85 ± 30.87c

## Discussion

### Effect of different planting densities and harvesting dates on the growth and development of *A. argyi*

Planting density is one of the most important factors affecting the growth and development of plants. Density determines the number of plants per unit area, and its variation affects the uptake of space, water, nutrients, and light, which in turn affects the outward form of the plant (Fang et al., 2018; Zhou et al., 2022). Inappropriate plant spacing may be harmful for plant growth, yield and quality (Souri & Hatamian, 2019). Excessive density results in poor ventilation, intense plant competition for light and nutrients and a low photosynthetic capacity of the population (Wang et al., 2020). As density was reduced in our study, *A. argyi* exhibited a decrease in plant height, leaf spacing and the height of withered leaves, a thickening of the stem, and an increase in crown width and the number of effective leaves in the main stem. Together, these indicate that, when planted at too high a density, *A. argyi* plants increase their plant height and reduce branching in order to capture more sunlight. The increase in individual plant biomass and the decrease in yield acre suggest that higher planting densities can increase population yields by compensating for lower individual plant yields, but also that excessive population densities can result in lower leaves not receiving light and wilting. This could lead to an overall decrease in the photosynthetic efficiency of the population and ultimately result in a serious waste of resources. Ma et al. (2020) investigated the effects of different planting densities on the yield of *A. argyi* during rhizome propagation and found that an appropriate reduction in density promoted the thickening of *A. argyi*, a reduction in leaf spacing, an increase in leaf size, a decrease in the rate of withered leaves, and an increase in the number of effective leaves in the main stem. Gu et al. (2016) investigated the effect of different planting densities and harvesting dates on the growth and yield of *Blumea balsamifera* and found that, with an increase in density, the height of *Blumea balsamifera*, the rate of withered leaves increased, and the yield increased, which aligns with the results of the present study.

Harvesting date is an important factor affecting the efficacy and yield of medicinal plants (Wang et al., 2020). Harvesting too early results in a low yield with low active ingredient content, but harvesting too late leads to an increase in crop cost. The results of this study showed that, with the delay of harvest date, the plant height of *A. argyi* increased, the crown width increased, the number of effective leaves on the main stem increased, the height of dead leaves increased, the drying ratio increased at first and then decreased, the yield increased, and the yield increased. The maximum yield was achieved on July 8th, but it did not differ significantly from those of June 22nd or June 30th, thus indicating that appropriately delayed harvesting can increase the yield of *A. argyi* leaves per unit area. Gu et al. (2016) found that, with the extension of the growth time, the plant height of *Blumea balsamifera* increased, as did the rate of dead leaves and the number of branches. Luo (2021) conducted a study on the effect of different harvest times on the growth of *A. argyi* and found that, from May to June, the plant height of *A. argyi*, the thickness of the stems, and the fresh weight of the leaves and the drying ratio all increased, which is consistent with the results of our study.

### Effect of different planting densities and harvesting dates on quality of *A. argyi* leaves and moxa

Density determines the absorption of light and nutrients by plants during their growth, and this in turn affects the quality of medicinal materials. Reasonably dense planting can achieve high yield while still ensuring quality. In this study, the volatile oil components of *A. argyi* showed a gradual increase as planting density decreased, potentially due to improved ventilation and light penetration at lower densities. This finding is consistent with the research results of Zhu & Wang (2015) and Gu et al. (2016). The moxa wool yield rate decreased with reduced planting density, possibly due to higher withered leaf rates in dense field populations because moxa wool production primarily depends on upper leaves. This observation aligns with Ma’s research findings (Ma et al., 2020). Moxibustion, a traditional Chinese medical therapy, achieves its therapeutic effects through the combined action of pharmacological components and thermal effects. This study found that the calorific value and total flavonoid content of moxa wool decreased with reduced planting density. However, Ma et al. observed an increase in flavonoid components with decreasing density, which might be explained by the screening process during moxa wool production that removes powder and residue containing substantial organic components (Ma et al., 2020).

The quality of medicinal materials is closely related to harvesting time, as intrinsic indicators gradually change throughout the growth cycle. In this study, the volatile oil content in *A. argyi* leaves initially increased but then decreased across all densities with delayed harvesting time, peaking one week after the Dragon Boat Festival. Zhang et al. (2016) observed a similar trend in volatile oil and components, though their peak occurred 1–2 weeks before the festival (Tan et al., 2008). Hu et al. (2016) found minimal differences in volatile oil content across different harvesting times, potentially due to variations in temperature, light, and soil fertility affecting biosynthesis and metabolite accumulation. The calorific value, total flavonoid content, and polyphenol content of moxa wool in this study all reached their maximum values within one week of the Dragon Boat Festival. Xue et al. (2019) reported peak levels of flavonoid and phenolic acid components on June 18th (day of the Dragon Boat Festival), showing slight variation from our findings, possibly due to their broader harvesting period. For Zou et al. (2023) the calorific value of moxa wool ranged from 3,920 to 4,039 cal/g, showing minimal difference from our results.

### Comprehensive analysis of *A. argyi* leaves quality at different planting densities and on different harvesting dates

Because it a leaf-based medicinal material, it is essential to consider the yield as well as the maintenance of quality when investigating the effects of planting density and harvesting period on *A. argyi* leaves. The conventional harvesting period around the Dragon Boat Festival spans several days, a relatively broad time frame. This study further refined the optimal harvesting window. Some quality indicators in this study showed a negative correlation with yield, as lower planting densities resulted in better ventilation and light penetration, leading to higher individual plant yield and superior quality. However, the reduced number of plants per unit area resulted in lower overall field production. To balance quality maintenance with high yield, this study followed Zheng’s methodology and employed principal component analysis and membership function analysis to evaluate different treatments (Zheng et al., 2021). Chang et al. (2020) found that the volatile oil content of *A. argyi* peaked around the Dragon Boat Festival, which is consistent with our findings. However, their study focused solely on volatile components and covered a broader harvesting period without detailed subdivision. In contrast, our research conducted a comprehensive analysis of various quality indicators and provided a more refined harvesting timeline. Based on volatile oil content, total flavonoid content, and tannin content, Hu et al. (2016) determined the optimal harvesting period for Qiai (a variety of *A. argyi*) to be early June, approximately one week after the Dragon Boat Festival, which aligned with our results. Finally, comparing different densities during the optimal harvesting period revealed that the 20 cm × 40 cm and the 20 cm × 50 cm densities exhibited superior quality indicators compared to the 20 cm × 20 cm and the 20 cm × 30 cm densities. Of the two superior densities, the 20 cm × 50 cm density showed lower yield. In terms of practical production factors, an excessively low density increases weed growth and raises manual weeding costs. Therefore, 20 cm × 40 cm was identified as the optimal planting density, with June 30th (one week after the Dragon Boat Festival) as the recommended harvesting time. Consistent with our findings, Zhang, Gui & Zhang (2023) suggested an optimal planting density of 105,000–120,000 plants/hectare.

## Conclusions

Moderate thinning of planting density can promote robust growth of *A. argyi* plants, thereby increasing the number of effective leaves on the main stem and enhancing the drying ratio of leaves, though it may result in reduced overall field yield. Delaying the harvest time can effectively increase the yield, but it gradually diminishes the yield-enhancing effect. Appropriate thinning and moderate delay in harvesting can promote the accumulation of volatile oils, eucalyptol and borneol in *A. argyi* leaves, though it may also lead to decreased calorific value, total flavonoid content, polysaccharide content, and polyphenol content in moxa wool. Based on our comprehensive analyses of volatile oil content, moxa yield rate, eucalyptol content, borneol content, drying ratio, and yield across different planting densities and harvesting periods, the optimal planting density for spring-grown *A. argyi* was determined to be 20 cm × 40 cm, with harvesting one week after the Dragon Boat Festival. These conditions yielded the best overall quality and relatively high production.

## Supplemental Information

10.7717/peerj.20565/supp-1 Supplemental Information 1 Effect of planting density and harvesting date on quality of *Artemisia argyi* Lowercase letters indicate a significant difference between different densities at the same harvesting dates (*p* < 0.05), and uppercase letters indicate a significant difference between different harvesting dates at the same density (*p* < 0.05).

10.7717/peerj.20565/supp-2 Supplemental Information 2 Raw data

## Additional Information and Declarations

### Data Availability

The following information was supplied regarding data availability: The raw measurements are available in the Supplementary File.

## References

[ref-1] Adnan NS, Wu Y, Javaid I, Mohsin T, Saqib B, Shafeeq UR, Abdul H, Saif A, Ma X, Saqer S, Alotaibi, Ahmed ES, Yang G (2021). Nitrogen and plant density effects on growth, yield performance of two different cotton cultivars from different origin. Journal of King Saud University—Science.

[ref-2] Chang Y, Xue Z, Yang G, Guo M, Zhang D, Zheng Y, Guo L (2020). Dynamic changes of volatile components of Qiai from different harvest time based on GC-MS and chemometrics analysis. China Journal of Chinese Materia Medica.

[ref-3] Chen Y (2011). Study on good agricultural practice and quality control of *Prunella vulgaris*.

[ref-4] China Pharmacopoeia Committee (2020). Chinese pharmacopoeia of People’s Republic of China.

[ref-5] Deng X, Han W (2020). Determination of tea polyphenols in green tea extracts by ferrous tartrate-standard curve method. Journal of Nanjing Tech University (Natural Science Edition).

[ref-6] Dou H, Feng W, Xiao H, Zhao C (2009). Effect of cultivated measure on yield and quality of the essential oil from peppermint (*Mentha piperita* L.). Journal of Northwest A & F University (Natural Science Edition).

[ref-8] Fang X, Li Y, Nie J, Wang C, Huang K, Zhang Y, Zhang Y, She H, Liu X, Ruan R, Yuan X, Yi Z (2018). Effects of nitrogen fertilizer and planting density on the leaf photosynthetic characteristics, agronomic traits and grain yield in common buckwheat (*Fagopyrum esculentum* M.). Field Crops Research.

[ref-7] Fang J, Wang C, Wang L, Shi W, Guo Y, Li Y, Qin M, Ma Q (2021). Comparative study on determination method of polysaccharide conten in Hongqu Fuling tablets. Lishizhen Medicine and Materia Medica Research.

[ref-9] Farhadi N, Souri MK, Palirezalu A (2013). Effect of sowing dates on quantity and quality of castor bean (*Ricinus communis* L.) under semi-arid condition in Iran. Zeitschrift Fur Arznei-Und Gewurzpflanzen.

[ref-10] Ghanbari M, Souri MK, Mirzaei HH, Omidbaigi R (2016). The essential oils composition in two populations of *Achillea wilhelmsii* C. Koch from Iran. Zeitschrift Fur Arznei-& Gewurzpflanzen.

[ref-11] Gu C, Wang H, Zhao Z, Liu H, Luo C, Li J, Luo F (2016). Effect of plant density and harvest on yield and quality in *Blumea balsamifera*. Journal of Chinese Medicinal Materials.

[ref-12] Hu J, Xia H, Guo S, Zhang R, Li L, Long W, Wan D (2016). Detection of essential oils, total flavonoids and tannins contents in qiai and determination of optimum harvest time. China Journal of Traditional Chinese Medicine and Pharmacy.

[ref-13] Kang NS, Lee JH (2011). Characterisation of phenolic phytochemicals and quality changes related to the harvest times from the leaves of Korean purple perilla (*Perilla frutescens*). Food Chemistry.

[ref-14] Liu X, Ou Q, Li Z, Zheng X, Xie M, Zhang X, Hu S (2023). Analysis on the quality of 34 local edible daylily germplasm. China Seed Industry.

[ref-15] Luo G (2021). Accumulative rule of nitrogen, phosphorus and potassium absorption and its relationship with quality of *Artemisia*.

[ref-16] Ma L, Chen C, Kang L, Miao Y, Fang Y, Guo L, Liu D, Huang L (2020). Effects of different planting density, leaf position and leaf age on growth and quality of *Artemisia argyi* var. argyi ‘Qiai’. China Journal of Chinese Materia Medica.

[ref-17] Ozkan G, Baydar H, Erbas S (2010). The influence of harvest time on essential oil composition, phenolic constituents and antioxidant properties of Turkish oregano (*Origanum onites* L.). Journal of the Science of Food and Agriculture.

[ref-18] Shah AN, Tanveer M, Abbas A, Yildirim M, Shah AA, Ahmad MI, Wang Z, Sun W, Song Y (2021). Combating dual challenges in maize under high planting density: stem lodging and kernel abortion. Frontiers in Plant Science.

[ref-19] Souri MK (2016). Plants adaptation to control nitrification process in tropical region; case study with *Acrocomia totai* and *Brachiaria humidicola* plants. Open Agriculture.

[ref-20] Souri MK, Hatamian M (2019). Aminochelates in plant nutrition; a review. Journal of Plant Nutrition.

[ref-21] Tan X, Li Q, Chen X, Wang Z, Shi Z, Bi K, Jia Y (2008). Simultaneous determination of 13 bioactive compounds in Herba *Artemisiae Scopariae* (Yin Chen) from different harvest seasons by HPLC-DAD. Journal of Pharmaceutical and Biomedical Analysis.

[ref-22] Tian H, Xiong H, Xiong J, Zhang H, Cai H, Liu Y (2015). Comprehensive evaluation of the production performance of 14 silage maize varieties by principal component analysis and subordinate function method. Acta Agriculturae Universitatis Jiangxiensis.

[ref-23] Wang Y, Liu C, Li Y, Gu T, Su Y (2020). Effects of sowing density on growth and yield of potato under drip irrigation. Journal of Drainage and Irrigation Machinery Engineering.

[ref-39] Wu J, Wan D, Jiang Y, Zhou S, Hu H (2020). Determination of combustion heat value of raw material moxa for moxibustion therapy. Shanghai Journal of Acupuncture and Moxibustion.

[ref-24] Xue Z (2020). Study on chemical constituents of Qiai in different harvest time.

[ref-25] Xue Z, Guo L, Guo M, Yang G, Zhang D, Guo OL, Zheng Y (2019). Study on difference of chemical constituents of Qiai in different harvest periods. China Journal of Chinese Materia Medica.

[ref-26] Yang S, Yang X, Huang J, Li L (2009). Effects of application of N, P and K and plant density on growth of *Artemisia Annua* and yield of *Artemisia*. China Journal of Chinese Materia Medica.

[ref-27] You S, He X, Long X, Liu X (2011). Antimicrobial activities of volatile oil from Folium *Artemisiae Argyi*. Journal of Traditional Chinese Veterinary Medicine.

[ref-28] Zhan Y, Liao X, Yang Y, Luo Y, Xu L (2021). Optimization of extraction process and component analysis of polyphenols from Wan’ai. Journal of Henan Agricultural University.

[ref-29] Zhang J, Gui L, Zhang X (2023). Research on green synergistic cultivation technology of *Artemisia argyi*. Modern Agricultural Science and Technology.

[ref-30] Zhang Y, Kang L, Zhan Z, Nan T, Li Y, Zhou A, Guo L (2016). Study on yield, main components and toxic components of volatile oil from *Artemisia argyi* Levl. et Vant. qvai. gathered in different growing period. Modernization of Traditional Chinese Medicine and Materia Medica-World Science and Technology.

[ref-31] Zhang Z, Zhao Y, Guo Y, Zhai Z, Deng S, Bu Y, Fu X, Zhao Z, Yang C (2007). Effect of three cultivation measures on yield of *Nepeta cataria*. Journal of Chinese Medicinal Materials.

[ref-32] Zhao OM, Ruan P, Wang X, Mei L, Zhang J, Wang H, Tu G (2013). Effects of different sowing densities on yield and quality of *Fagopyrum dibotrys*. Hunan Agricultural Sciences.

[ref-33] Zheljazkov VD, Cantrell CL, Astatkie T, Hristov A (2010). Yield, content, and composition of peppermint and spearmints as a function of harvesting time and drying. Journal of Agricultural and Food Chemistry.

[ref-34] Zheng J, Li X, Ren S, Yang GT (2021). Effects of nitrogen fertilizer and potassium fertilizer combined with topdressing on yield and medicinal components content of the second, third and last stubble flower of honeysuckle (*Lonicera japonica* Thunb). Forestry and Ecological Sciences.

[ref-35] Zheng K, Zhong X, Zhang H (2020). Advances in research on constituents and pharmacological effects of *Artemisia argyi* essential oil. Chinese Journal of Experimental Traditional Medical Formulae.

[ref-36] Zhou W, Li M, Tan X, Wang Y, Wang H, Jiang X, Duan Y, Zhang M (2022). Effects of sowing density on growth, nutritional quality and soil enzyme activity of *Pinellia ternata* in different seasons. Crops.

[ref-38] Zhu X, Wang W (2015). Comparison of content of eucalyptol in Artemisia argyi of 5 different planting densities. China Pharmacy.

[ref-37] Zou R, Jiang H, Yang H, Yu G, Guan G (2023). Research on quality evaluation criteria based on combustion characteristics of moxa wool. Lishizhen Medicine and Materia Medica Research.

